# Imbalance in the game of T cells: What can the CD4/CD8 T-cell ratio tell us about HIV and health?

**DOI:** 10.1371/journal.ppat.1006624

**Published:** 2017-11-02

**Authors:** Joseph A. McBride, Rob Striker

**Affiliations:** 1 William S. Middleton Memorial Veterans Hospital and the Department of Medicine, Division of Infectious Disease, Madison, Wisconsin, United States of America; 2 Department of Medicine, Division of Infectious Disease, University of Wisconsin-Madison, School of Medicine and Public Health, Madison, Wisconsin, United States of America; 3 Department of Pediatrics, Division of Infectious Disease, University of Wisconsin-Madison, School of Medicine and Public Health, Madison, Wisconsin, United States of America; University of Pittsburgh, UNITED STATES

## Introduction

Antiretroviral treatment (ART) has transformed human immunodeficiency virus (HIV) from a deadly disease to a chronic illness that potentially has little effect on life expectancy. Modern ART can eliminate viremia and lower the risk of transmission. National data from the United States demonstrate that 81% of infected individuals receiving ART are virally suppressed [1]. With treatment available, HIV morbidity and mortality are not determined by opportunistic infections or AIDS-defining illnesses but rather by non-AIDS–defining conditions, including cardiovascular disease, liver disease, kidney disease, malignancies, neurocognitive disorders, and even autoimmune diseases [2]. To some, autoimmunity coexisting with HIV may be surprising; however, its presence illustrates how HIV’s immunopathology is more consistent with immune dysfunction than immune suppression alone.

HIV viral load and the resulting decrease in absolute CD4 T cells have historically served as biomarkers for HIV’s immune suppression and response to treatment. However, with successful modern ART and viral suppression, absolute CD4 count and HIV viral load may not accurately reflect the risks facing patients because immune dysfunction persists despite normalization of CD4 counts [3]. One explanation is that these markers fail to truly describe HIV’s overall immune dysfunction contributing to today’s morbidity and mortality. The CD4/CD8 ratio more accurately describes this overall immune dysfunction and may be a better biomarker for disease progression, response to treatment, morbidity, and mortality for the virally suppressed. A greater understanding of the CD4/CD8 ratio and the impact of its manipulation should be a target for future HIV research.

## What is the CD4/CD8 ratio?

CD4 helper/inducer cells and CD8 cytotoxic/suppressor cells are 2 phenotypes of T lymphocytes, characterized by distinct surface markers and functions that mostly reside in lymph nodes but also circulate in the blood. The normal CD4/CD8 ratio in healthy hosts is poorly defined. Ratios between 1.5 and 2.5 are generally considered normal; however, a wide heterogeneity exists because sex, age, ethnicity, genetics, exposures, and infections may all impact the ratio [4–7]. Normal ratios can invert through isolated apoptotic or targeted cell death of circulating CD4 cells, expansion of CD8 cells, or a combination of both phenomena. A low or inverted CD4/CD8 ratio is an immune risk phenotype and is associated with altered immune function, immune senescence, and chronic inflammation in both HIV-infected and uninfected populations [8–11].

The prevalence of an inverted CD4/CD8 ratio increases with age. An inverted ratio is seen in 8% of 20- to 59-year-olds and in 16% of 60- to 94-year-olds [7]. Women across all age groups are less likely to have an inverted ratio than their male counterparts [7]. Age- and hormone-related atrophy of the thymus is theorized to explain the differences between populations. Hormonal influence on the ratio is supported by a correlation between low plasma estradiol levels, high circulating CD8, and low CD4/CD8 ratios in women with premature ovarian failure [12]. Mouse models further highlight the importance of age and estrogen on the CD4/CD8 ratio because lower ratios are reported in mice following both natural menopause and ovariectomy [13]. Persistence of the thymus is associated with better ratio recovery in HIV treatment [14].

## Are abnormal CD4/CD8 ratios associated with pathology in the HIV negative population?

In the HIV negative population, a low CD4/CD8 immune risk phenotype reflects immune senescence, is associated with wide-ranging pathology, and may also predict morbidity and mortality [7,15–22]. Irreversible disruption of self-immunologic tolerance to endogenous antigens is a hallmark of autoimmune disease. In this setting of immune dysfunction, an abnormal CD4/CD8 ratio can emerge. Furthermore, while an abnormal ratio is not uniformly present in all autoimmune diseases, a decreased CD4/CD8 ratio is consistently seen in systemic lupus erythematosus [15–17]. A low CD4/CD8 ratio reflects β-cell destruction and may predict diabetes diagnoses in first-degree relatives of type 1 diabetic probands [18]. In a population study of solid neoplasms, an inverted CD4/CD8 ratio is associated with metastatic disease as compared with cancer patients without metastasis [19]. Moreover, following acute myocardial infarction and cardiopulmonary resuscitation, a fixed low CD4/CD8 ratio is a poor prognostic sign [20]. Despite these associations, it is important to acknowledge that the presence of a low CD4/CD8 ratio is not clearly the cause or the effect of the above pathology. This acknowledgment is further highlighted by the presence of a low ratio in conditions outside the umbrella of traditional organic pathology, including an association between low ratios and pessimists [21].

Conflicting literature exists regarding the use of an inverted CD4/CD8 ratio (<1.0) as a predictor for mortality in elderly HIV-negative populations. Two longitudinal cohorts of elderly Swedish individuals demonstrated that an inverted ratio (<1.0) was associated with frailty and mortality [7,10]. These studies helped define the immune risk phenotype and raised the possibility of using the CD4/CD8 ratio as a biomarker to stratify risk in elderly populations. Later cohort studies in Spain and the United Kingdom found that while a low CD4/CD8 ratio was associated with time to death in unadjusted analyses, no association between the ratio and morbidity was found in multivariable analyses [22,23]. Moreover, a recent cross-sectional study of frailty and prospective cohort study of morbidity in residents of Canadian nursing homes found that greater percentages of central memory CD8^+^ T cells were more predictive of increased frailty than other immune phenotypes, including an inverted CD4/CD8 ratio [24]. Thus, the CD4/CD8 ratio may not be a marker for morbidity and/or mortality in all populations.

## Why should CD4/CD8 ratio be used as a marker in the HIV population?

The natural history of untreated HIV infection has opposing effects on circulating CD4 and CD8 T lymphocytes. Before HIV lowers CD4 cells, circulating CD8 cells will typically rise in response to the infection, resulting in a low CD4/CD8 ratio [25]. In the setting of ART, some patients will restore CD4 counts and experience a decline in CD8 counts, leading to normalization of the ratio. For other individuals, however, despite suppression of the virus and improvement of CD4 levels, the high levels of circulating CD8 cells are maintained, and their ratios fail to improve [11,26,27]. A recent cross-sectional study of 334 of these virologically suppressed patients demonstrates that a lower CD4/CD8 ratio during treatment predicts residual HIV viremia (≥1 copy/ml), as detected by single-copy assay [28]. Whether this residual viremia is a cause or effect of a lower CD4/CD8 ratio is unknown, but the association highlights the discordant immune activation and immune senescence in the virologically suppressed.

Today, more researchers are investigating the ratio’s utility as a biomarker and are examining the link between the CD4/CD8 ratio and outcome in the HIV-positive population [29,30]. A low ratio, and not the absolute CD4 count, is the primary factor associated with a lack of desired response following hepatitis B and yellow fever vaccination [31,32]. Low ratios have been tied with HIV and the development of neurocognitive disorders, lung cancer, and chronic obstructive pulmonary disease, while elevations of activated CD8 cells are linked to myocardial infarction [33–36]. Further evidence demonstrates an overall increased risk of morbidity and mortality in HIV-positive individuals who fail to normalize their ratio [11,36]. The ratio is independently associated with markers of age-associated disease, including carotid intima-media thickness, arterial stiffness, glomerular filtration rate, and sarcopenia [9]. These studies demonstrate similar evidence of altered immune function and chronic inflammation as seen in the noninfected elderly cohorts; however, in the HIV-positive population, the immune activation and senescence are seen at a much younger age [9]. Although the link between low ratio and poor outcomes is growing, not all studies agree. A recent observational cohort of virologically suppressed individuals failed to replicate evidence that the ratio was prognostic for non-AIDS mortality but found that both low CD4/CD8 ratio and high CD8 count were associated with excess AIDS mortality in an HIV population that was otherwise healthy [37].

## What alters the CD4/CD8 ratio, and do other pathogens aside from HIV change it?

Untreated HIV infection drives the CD4/CD8 ratio lower. In some populations, initiation of ART can increase the ratio; however, early and continuous treatment is essential. If ART begins during primary HIV infection, 90% of patients will achieve normalization of their CD4/CD8 ratio within 6 years of antiviral therapy, and almost all will normalize within a decade [27]. Conversely, if ART begins during chronic HIV, then the majority of patients will fail to normalize their ratio even after 14 years of viral suppression and restoration of CD4 levels to >500 [27]. Treatment interruptions are also deleterious to the ratio, so early initiation of ART and continuous adherence to therapy should be stressed [38].

The optimal treatment regimen for ratio normalization is unknown. Integrase inhibitor, rather than nonnucleoside reverse-transcriptase inhibitor–or protease inhibitor–based regimens, is theorized to best improve immune dysfunction. Faster CD4/CD8 ratio normalization with raltegravir- versus efavirenz-based regimens supports the claim of integrase inhibitor superiority [39]. The impact of newer integrase inhibitors such as dolutegravir and elvitegravir is not known.

Cytomegalovirus (CMV) infection has a significant impact on the CD4/CD8 ratio in both the HIV-positive and -negative populations through the expansion of CMV-specific CD8 cells. This accumulation of CMV-specific CD8 cells lowers CD4/CD8 ratios, leading to the immune risk phenotype [40]. In HIV-uninfected populations, these clonal expansions are evident in the elderly; however, in the HIV-infected population, these CMV-specific CD8 clonal expansions are seen at a younger age [41,42]. Whether other chronic infections such as tuberculosis, dimorphic fungi, toxoplasmosis, or leishmaniasis also lead to CD8 expansion with altered ratios is less well studied but likely occurs to a greater degree in HIV-infected than -uninfected individuals.

CMV coinfection could represent a potential therapeutic target for manipulating the ratio. In HIV and CMV coinfection, persistent low levels of CMV replication are associated with lower CD4/CD8 ratios both at diagnosis and while on ART [43]. Moreover, reductions of activated CD8 T cells are seen in the setting of short-term CMV treatment with valganciclovir in the coinfected [44]. The impact of long-term simultaneous treatment of HIV and CMV on immune senescence, the CD4/CD8 ratio, and overall morbidity is not known. Data on the effect of pathogens and treatment (including immunotherapy) on T-cell subsets are likely generated by many investigators in the *PLOS Pathogens* community but are not often included in final publications.

## Could the CD4/CD8 ratio serve as a marker for the HIV reservoir?

Early, effective, and uninterrupted ART improves the CD4/CD8 ratio. Early ART is also shown to reduce the size of the HIV reservoir [27,44]. Therefore, the use of the CD4/CD8 ratio as a peripheral surrogate of the HIV reservoir is a hypothesis worthy of investigation. Researchers have linked the CD4/CD8 ratio with integrated levels of HIV–DNA in peripheral blood cells [27,45]. Similarly, an inverse correlation is demonstrated between CD4/CD8 ratio and the frequency of CD4 T cells carrying HIV–proviral DNA [45]. Furthermore, lower ratios during ART are also associated with persistently higher HIV–DNA despite measurable HIV–RNA suppression [46]. While raising the ratio above 1.0 is likely a prerequisite or an associated phenomenon with reservoir reduction, a high ratio alone is likely insufficient to eradicate the reservoir, particularly in older patients with late initiation of treatment. If a sturdier relationship between ratio and reservoir can be proven, then therapies aimed at reducing the size of the viral reservoir may use the ratio for assessing their success.

## Conclusion

Viral suppression and CD4 response will always remain important treatment goals in HIV management. Yet if treatment success is defined by these parameters alone, then we may fail to recognize certain risks encountered by today’s HIV population. Evidence exists to consider the CD4/CD8 ratio a biomarker for assessing risks facing the modern aviremic HIV population. Yet the impact of other immune stimuli on the ratio, particularly non-HIV drugs and copathogens, is often unknown. We plan to highlight this research as it becomes available at www.GameofTcells.medicine.wisc.edu.

Manipulation of the ratio could serve as a potential target for further HIV therapeutic interventions, and measurement of the ratio may serve as an adequate surrogate for the HIV reservoir. More knowledge is needed regarding the impact of specific ART regimens and the simultaneous treatment of coinfections. Immunotherapy as treatment for oncologic disorders is increasing, yet surprisingly, any impact of immunotherapy on the ratio is not routinely reported. Researchers using human and nonhuman animal models should consider using the CD4/CD8 ratio as a marker in their investigations of HIV and other chronic conditions that can facilitate translation into clinical practice.
